# Bi-axial grown amorphous MoS_x_ bridged with oxygen on r-GO as a superior stable and efficient nonprecious catalyst for hydrogen evolution

**DOI:** 10.1038/srep41190

**Published:** 2017-01-20

**Authors:** Cheol-Ho Lee, Jin-Mun Yun, Sungho Lee, Seong Mu Jo, KwangSup Eom, Doh C. Lee, Han-Ik Joh, Thomas F. Fuller

**Affiliations:** 1 Carbon Convergence Materials Research Center, Institute of Advanced Composite Materials, Korea Institute of Science and Technology (KIST), chudong-ro 92, Bongdong-eup, Wanju, Jeollabukdo 55324, Republic of Korea; 2 Department of Chemical and Biomolecular Engineering (BK21+ Program), KAIST Institute for the Nanocentury, Korea Advanced Institute of Science and Technology (KAIST), Daejeon 34141, Republic of Korea; 3 Radiation Research Division for Industry and Environment, Korea Atomic Energy Research Institute (KAERI), Geumgu-gil 29, Jeongeup-si, Jeollabuk-do 56212, Republic of Korea; 4 Department of Materials Science and Engineering, Gwangju Institute of Science and Technology (GIST), Gwangju, 61005, Republic of Korea; 5 School of Chemical & Biomolecular Engineering, Georgia Institute of Technology, Atlanta, Georgia 30332, USA

## Abstract

Amorphous molybdenum sulfide (MoS_x_) is covalently anchored to reduced graphene oxide (r-GO) via a simple one-pot reaction, thereby inducing the reduction of GO and simultaneous doping of heteroatoms on the GO. The oxygen atoms form a bridged between MoS_x_ and GO and play a crucial role in the fine dispersion of the MoS_x_ particles, control of planar MoS_x_ growth, and increase of exposed active sulfur sites. This bridging leads to highly efficient (−157 mV overpotential and 41 mV/decade Tafel slope) and stable (95% versus initial activity after 1000 cycles) electrocatalyst for hydrogen evolution.

Hydrogen is becoming increasingly essential as an environmentally friendly energy carrier. Global warming and abnormal climate resulting from the anthropogenic use of petroleum and environmental pollution accelerated its importance. Hence, hydrogen should be produced renewably, using clean technologies rather than by the steam reforming of natural gas. Among the potential technologies, sustainable hydrogen production using an electrochemically driven water dissociation process has been intensively explored. Efficient catalysts for the electrochemical hydrogen evolution reaction (HER) are needed to reduce the overpotential and increase the efficiency of hydrogen production. Platinum (Pt) shows the best electrocatalytic activity for the HER in acidic media; however, Pt is too expensive to be used beyond a few specialized applications. Hence, replacing Pt with low-cost and earth abundant materials for electrocatalysts is a critical challenge^1^. In-depth research seeking highly efficient and stable HER catalysts has become necessary, though various materials such as metal dichalcogenides, polymer-based carbon nitride, transition metal carbides, and nickel alloys have been proposed as promising catalysts^2^,^3^,^4^,^5^.

Recently, a family of two-dimensional transition metal disulfides (TMDs) with MS_2_ structure, where M is a transition metal such as molybdenum (Mo) or tungsten (W) and S is sulfur, has attracted much attention. These materials are a promising class of HER catalyst because they are one of the most efficient materials among the nonprecious catalysts. It is well known that the efficient electrochemical activity of the TMD stems from the S-terminated edge or strained metallic phase of MS_2_, while the basal plane of semiconducting MS_2_ is catalytically inert^6^,^7^,^8^. Jaramillo *et al*. reported that only one in four atoms of MoS_2_ edge sites could evolve H_2_ molecules because of the atomic hydrogen coverage of only 25% on the edge in contrast to Pt (111) as calculated by Density Functional Theory^9^. Hence, it is necessary either to synthesize nano-sized particles or to tune the electronic structure of the edges to improve the activity. However, unsupported nanoscale MoS_2_ with a large number of edge sites is thermodynamically unstable, leading to aggregation or transformation of the nanoparticles^10^. In addition, S-terminated edges are easily oxidized in acidic media^11^. These intrinsic properties induce the deactivation and instability of the materials when used for HER. There are two strategies to overcome these challenges: (1) controlling the morphology and (2) designing hybrid structure. Specific morphology control has been achieved using hard or soft templates such as MoO_3_/MoS_2_ (core/shell) nanowire, highly ordered double-gyroid MoS_2_, vertically aligned MoS_2_, and MoS_2_ flowers^12^,^13^,^14^,^15^. Although these methods could prevent the degradation of MoS_2_ electrochemical activity, the synthetic processes are unsuitable for the industrial scale because of their complexity and expense. Meanwhile, the hybrid structure consists of carbon supported MoS_2_, which has exhibited a strong interaction between the TMD and the carbon support, minimizing the thermodynamically unstable properties and improving the morphological and electrochemical stability. However, much remains to be studied regarding the origins of the interaction and the properties of the carbon supported amorphous molybdenum sulfide. Herein, we report a one-pot synthetic strategy to produce the highly-stable and efficient MoS_x_/r-GO catalyst via oxygen bridging between amorphous MoS_x_ and r-GO. These features are induced by the functional coupling of oxygen bridges between molybdenum sulfide and graphene oxide as shown in Fig. 1a.

## Results

The molybdenum sulfide catalysts were easily synthesized by the wet-chemical reaction of (NH_4_)_2_MoS_4_ and HCl in an aqueous dispersion of graphene oxide (GO) at room-temperature. The precursor was reduced to molybdenum sulfide (MoS_x_, sulfur content (x) changed from 1 to 3) on the graphene supports. To investigate the effects of the amount of deposited MoS_x_ particles on electrochemical hydrogen production, we synthesized the MoS_x_/r-GO catalysts with the amount of MoS_x_ precursor varying from 0.1 to 0.7 g. (Hereafter, a catalyst prepared using y g of MoS_x_ precursor and a fixed amount of GO is denoted as y MoS_x_/r-GO sample.) For comparison, unsupported MoS_3_ particles were also prepared in the absence of GO using the same process.

**Figure 1:**
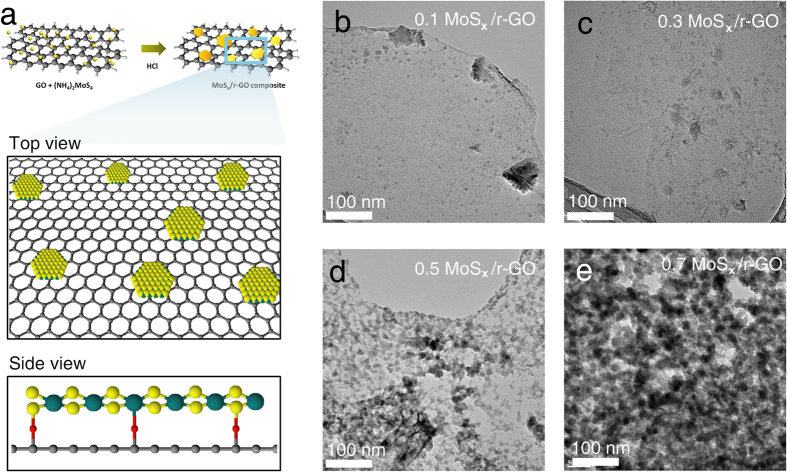
Synthesis of MoS_x_/r-GO composites. (**a**) Schematic synthesis process and internal structure of MoS_x_/r-GO at the top and side position. (**b**–**e**) TEM images of MoS_x_/r-GO composites by added amount of (NH_4_)_2_MoS_4_ precursor: (**b**) 0.1, (**c**) 0.3, (**d**) 0.5, and (**e**) 0.7 g.

High-resolution transmission electron microscopy (HR-TEM) images show that both the size and the amount of MoS_x_ particles on thin graphene flake depend strongly on the weight of precursor used as shown in Fig. 1b–e. For 0.5 MoS_x_/r-GO, the particles are uniformly deposited on the GO surface with full coverage, whereas catalysts synthesized with either less or more than 0.5 MoS_x_/r-GO exhibited insufficient or aggregated particle features, respectively. However, the particle size of MoS_x_/r-GO is relatively smaller than for unsupported MoS_3_ (see Supplementary Figure S1). Elemental mapping was conducted using energy dispersive spectroscopy (EDS) to confirm the origin of the particles deposited on the r-GO sheets. For all MoS_x_/r-GO composite samples, the positions of Mo atoms are highly correlated with the positions of S atoms, and MoS_x_ particles are considered to have been successfully synthesized on the r-GO sheets (see Supplementary Figure S2).

**Figure 2:**
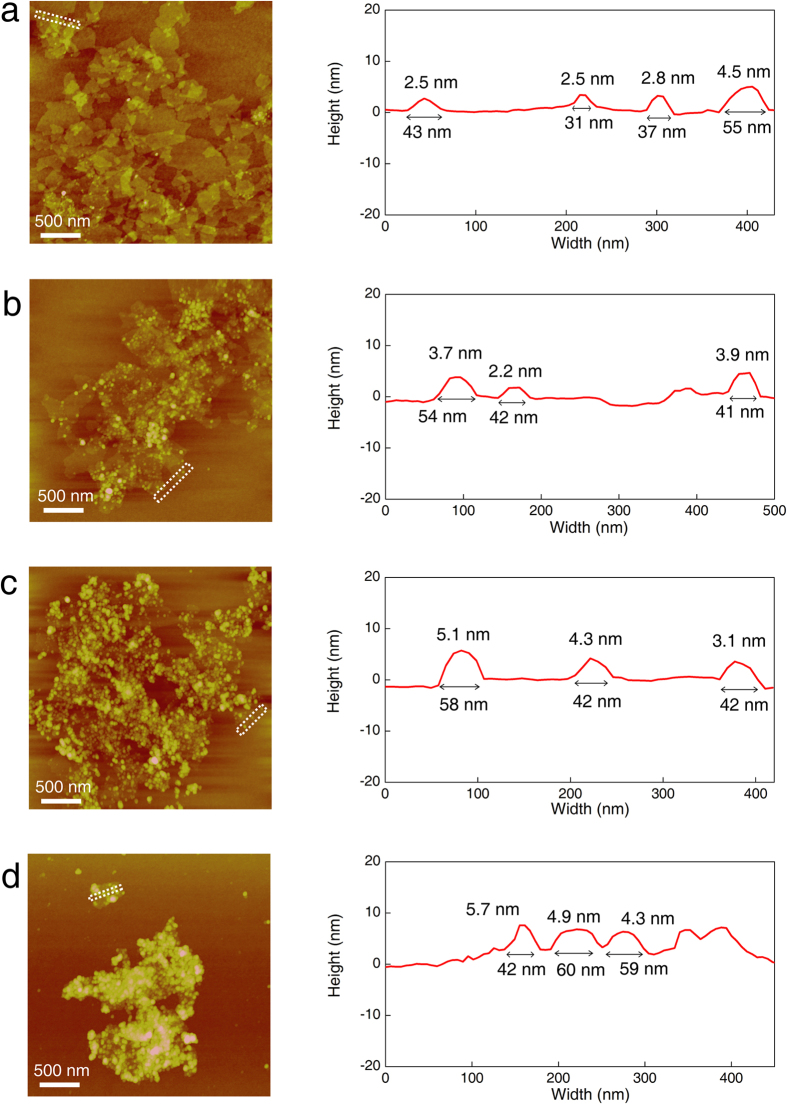
AFM images of as-synthesized MoS_x_/r-GO composites by added amount of (NH_4_)_2_MoS_4_ precursor. (**a**) 0.1, (**b**) 0.3, (**c**) 0.5, and (**d**) 0.7 g, and corresponding height spectra along with dashed rectangles in AFM images indicating that MoS_x_ on r-GO has a planar shape with a high aspect ratio.

We further investigated the morphological features of as-synthesized MoS_x_/r-GO composites using atomic force microscopy (AFM) as shown in Fig. 2. Each GO sheet has a thickness of ~1.1 nm consistent with double- or triple-layered GO. Similar to the TEM results for the MoS_x_/r-GO composites, the width and height of MoS_x_ on the r-GO composites depend on the amount of MoS_x_ precursor. The average width of the MoS_x_ particles on r-GO were increased from 50.2 nm to 58.8, 66.8, and 87.6 nm for 0.1, 0.3, 0.5, and 0.7 MoS_x_/r-GO, respectively. Average thickness also gradually increased from 2.9 nm to 4.3, 5.6, and 7.7 nm for 0.1, 0.3, 0.5, and 0.7 MoS_x_/r-GO respectively. The unsupported MoS_3_ particles synthesized using the same method show a larger average particle width of 117.8 nm and height of 22.3 nm (see Supplementary Figure S3). Interestingly, all the MoS_x_/r-GO composites show a very high aspect ratio–above 10, in contrast to the unsupported MoS_3_. This result indicates that MoS_x_ particles were biaxially grown on the r-GO surface in a planar or coin shape. Thus, we believed that interaction between the precursor and the GO might affect the growth and morphology of the particles, which are closely related to the catalytically active edge sites^16^.

**Figure 3:**
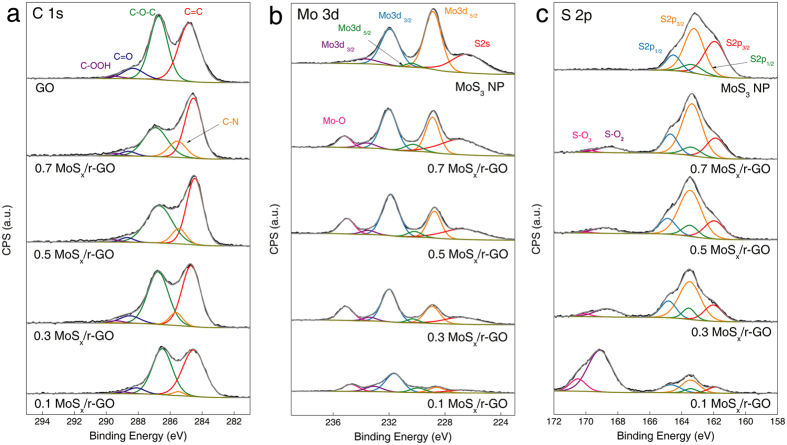
XPS spectra and fitted peaks of MoS_x_/r-GO composites. (**a**) C1s spectra shows reduction of GO with increasing precursor. (**b**) Fitted Mo3d peaks indicate Mo with both 4+ and 5+ oxidation states. (**c**) Deconvolution of S2p consisting of both terminal S and bridging S.

The structural composition and interaction between the MoS_x_ and r-GO were investigated using X-ray photoelectron spectroscopy (XPS) as shown in Fig. 3. The XPS spectra of all composite samples exhibited predominant C1s, Mo3d, and S2p peaks. The peak intensities of oxygen functional groups on the GO, such as epoxy, carbonyl, and carboxyl groups, which are observed at binding energies (BE) of 286.7, 288.4, and 289.5 eV, respectively, gradually decreased with increasing precursor concentrations (Fig. 3a). Thus, it is believed that the insulating GO substrate can be spontaneously reduced to the conductive r-GO by the hydrazine or ammonium chloride species generated during the growth reaction of MoS_x_ particles^17^. Similar trends were observed in the X-ray diffraction (XRD) patterns of the GO and MoS_x_/r-GO composites as shown in Figure S4. The sharp (001) peak of GO at 10.8 degrees, which represents the wider interlayer distance between the graphitic layers compared to graphite, was shifted into the (002) plane at 22.0 degrees for the composites.

Remarkably, the stoichiometric S/Mo ratios of the composite gradually increased from 1.5 to 2.3, 2.6, and 3.3 for 0.1, 0.3, 0.5, and 0.7 MoS_x_/r-GO, respectively, while the ratio of unsupported MoS_3_ particles was approximately ~3.0, indicating the MoS_3_ structure. The Mo 3d spectrum with Mo 3d^3/2^ and Mo 3d^5/2^ doublets indicates that the Mo metal in all composite samples had the 4+ oxidation state. In particular, Mo3d^3/2^ (233.2 eV) and Mo3d^5/2^ (230.0 eV) in the composite samples were observed at higher BE than in unsupported MoS_3_ (231.5 eV and 228.4 eV for Mo3d^3/2^ and Mo3d^5/2^, respectively). These values can be attributed to the presence of Mo^5+^ and indicate that each of the Mo atoms in the composites was randomly bonded with 2 ~ 3S atoms as indicated in the stoichiometric S/Mo ratio. Wang *et al*. reported that MoS_x_ was a fundamentally and thermodynamically amorphous structure with many active edge sites, in contrast to crystalline MoS_2_, when the stoichiometric ratio of S atoms to Mo atoms is above 2^18^. There are broad diffraction peaks in all our MoS_x_ and MoS_3_ particles in XRD patterns of Figure S4. Thus, the resulting MoS_x_ particles have an amorphous structure irrespective of the GO, which is expected to expose more active edge sites of MoS_x_.

The S 2p spectrum consists of two doublets. One doublet with higher BE (S2p^3/2^ = 163.2 eV and S2p^1/2^ = 164.6 eV) is attributed to the existence of both bridging S_2_^2−^ and/or apical S^2−^ ligands. The other doublet with relatively lower BE (S2p^3/2^ = 162.0 eV and S2p^1/2^ = 163.2 eV) stems from the existence of the terminal S_2_^2−^ and/or S^2−^ 19. Considering the previous reports that the HER activity of MoS_x_ is highly correlated to the amount of terminated S-edge sites, it can be expected that the abundance of catalytic edge sites estimated from the deconvolution of S peaks (area ratio of edged versus bridged S = ~5/4) has a beneficial effect on the hydrogen evolution efficiency.

Importantly, new peaks related to molybdenum-oxygen (Mo-O) bonding (235.6 eV) and sulfur-oxygen (S-O) bonding (169.2 and 170.5 eV) are observed only for the MoS_x_/r-GO composite in the presence of graphene oxide. The amount of S-O bonding, both the strong SO_2_ and weak SO_3_ configurations, significantly decreases with increasing precursor concentration, while Mo-O bonding (MoO) increases slightly with precursor concentration. In particular, in the case of 0.5 MoS_x_/r-GO, 12.4 and 7.3 atomic percent of the molybdenum and sulfur atoms, respectively, in the edge area of MoS_x_ particles are covalently bonded to oxygen functional groups on r-GO sheets^20^. Therefore, we believe that most of the epoxide among the oxygen functional groups plays a crucial role in the anchoring or bridging between MoS_x_ and GO. From the XPS analysis, we can conclude that the novel oxygen-bridged structure could induce the modulation of particle growth, Mo/S stoichiometry, and an amorphous configuration with more exposed active sites, which are expected to improve catalytic activity for hydrogen evolution.

**Figure 4:**
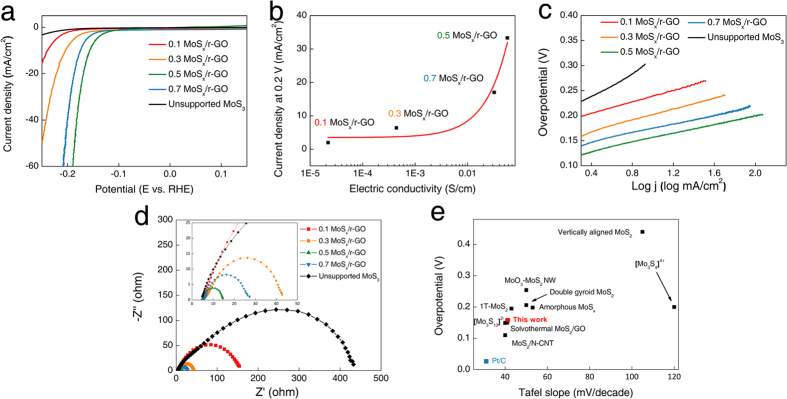
(**a**) Polarized curves of each MoS_x_/r-GO catalysts and unsupported MoS_3_, (**b**) relationship between electrical conductivity and HER activity. (**c**) The corresponding Tafel plot obtained from polarized curve. (**d**) Nyquist plots measured at 0.2 V. (**e**) Overpotential and Tafel slope of various molybdenum sulfide catalysts.

We investigated the electrochemical HER performance of MoS_x_/r-GO composites deposited on a glassy carbon electrode in 0.5 M H_2_SO_4_ aqueous electrolyte using a typical three electrode setup as shown in the polarization curves (J-V) of current density (J) plotted against potential (V) of Fig. 4a. The overpotentials of all MoS_x_/r-GO composites at 10 mA/cm^2^ were −240, −204, −157, and −176 mV for 0.1, 0.3, 0.5, and 0.7 MoS_x_/r-GO respectively. The activity of composite catalysts in the precursor range from 0.1 to 0.5 g dramatically improved from J = −1.5 to −59.7 mA/cm^2^ at 190 mV (vs RHE). On the other hand, the HER activity of the 0.7 MoS_x_/r-GO catalyst was comparatively decreased, probably due to decreased electrical conductivity and reduced catalytically active sites^21^. The conductivity of 0.5 MoS_x_/r-GO (5.7 × 10^−2^ S/cm) measured by the 4-point probe resistivity measurement was three and two orders higher in magnitude than the conductivity of 0.1 and 0.3 MoS_x_/r-GO, respectively. The increased conductivity would be originated from the reduction of GO. At the same time, 0.7 MoS_x_/r-GO had a 44% lower value of 3.2 × 10^−2^ S/cm, with respect to the relatively low amount of r-GO even though the high degree of reduction. Therefore, we concluded that the current density for the HER is closely related to the conductivity as shown in Fig. 4b; and thus high electrical conductivity would mainly affect the improvement of electrochemical HER activity.

To confirm the quantitative catalytic activity and rate determining step (RDS), we fitted a Tafel plot based on the HER polarization curves as shown in Fig. 4c. The calculated Tafel slopes were 54, 53, 42, 41, and 112 mV/decade for 0.1, 0.3, 0.5, 0.7 MoS_x_/r-GO, and unsupported MoS_x_ particles, respectively. The possible HER process in acidic electrolyte generally consists of three steps; Volmer (H^+^ + e^−^ → H_ads_, <120 mV/decade), Heyrovsky (H_ads_ + h^+^+e^−^ → H_2_, <40 mV/decade), and Tafel (H_ads_ + H_ads_ → H_2_, <30 mV/decade)^22^. Considering the Tafel slopes of the catalysts, both the unsupported MoS_x_ and the MoS_x_/r-GO in this study might favor an electrochemical desorption mechanism, in which electrochemical desorption is the RDS, although the inherent mechanism of Mo sulfide based catalysts has been inconclusive to date^16^. However, the resulting Tafel slope of 0.5 MoS_x_/r-GO is the smallest among the catalysts. Previous studies have reported that the major factors affecting the HER activity are the surface energy for hydrogen desorption and the rate of electron transfer^23^. It is well-known that MoS_x_ itself is a semiconducting material, while the surface energy of MoS_x_ is theoretically limited to desorbing the hydrogen^23^. Thus, it can be concluded that obvious differences in the HER activity of the catalysts attributed to the electron transfer are evident in the electrochemical impedance spectra at 0.2 V (vs RHE). The MoS_x_/r-GO catalysts, especially 0.5(~14.6 Ω), show far lower charge-transfer impedance than unsupported MoS_x_ (~432.6 Ω), leading to higher HER activity (Fig. 4d). In addition, calculated active site and turnover frequency (TOF) of 0.5 MoS_x_/r-GO at 0.1 V were 2.11 * 10^14^ Mo atoms/cm^2^ and 4.8 s^−1^, respectively. The values of MoS_x_/r-GO are similar value compared to other studies^19^,^24^. The resulting overpotential and Tafel slope of 0.5 MoS_x_/r-GO are among of the best values among the recently published studies on materials such as highly conductive molybdenum sulfides (1T-MoS_2_, MoS_x_/N-CNT, solvothermal MoS_2_, and [Mo_3_S_13_]^2−^) and conventional molybdenum sulfides with no conductive substrate (double gyroid MoS_2_, vertically aligned MoS_2_, MoO_3_-MoS_2_ nanowire (NW), amorphous MoS_x_, and [Mo_3_S_4_]^4−^) as shown in Fig. 4e^12^,^13^,^14^,^16^,^19^,^25^,^26^,^27^,^28^. The achieved performance for hydrogen production is significantly useful compared to the materials for solar hydrogen production^29^,^30^.

**Figure 5:**
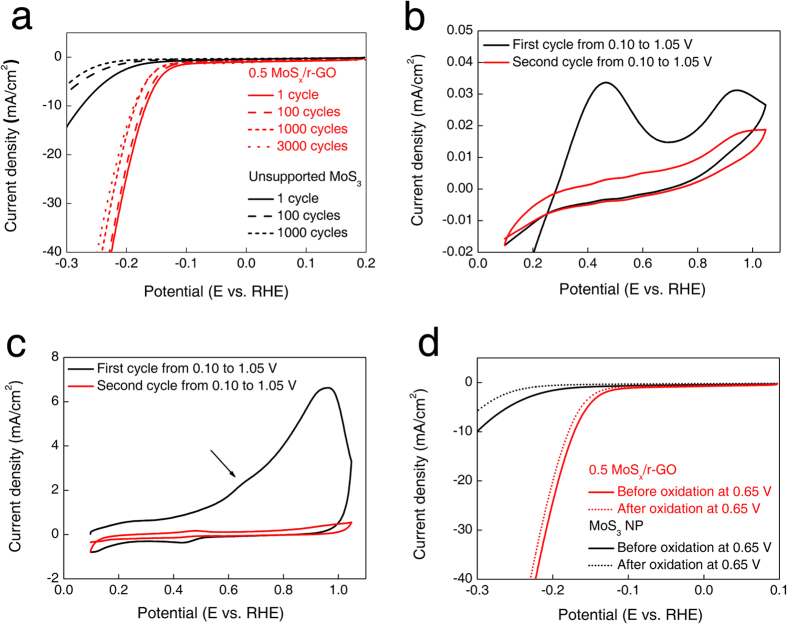
(**a**) Catalytic stability of 0.5 MoS_x_/r-GO composite for 3,000 cycles and unsupported MoS_3_ for 1,000 cycles. Cyclic voltammetry of (**b**) unsupported MoS_3_ and (**c**) 0.5 MoS_x_/r-GO from 0.10 to 1.05 V. (**d**) HER activity of unsupported MoS_3_ and 0.5 MoS_x_/r-GO before/after electrochemical oxidation at 0.65 V.

The catalytic stability of 0.5 MoS_x_/r-GO over 3,000 cycles was measured by cyclic voltammetry with a potential range from −0.3 to 0.2 V as shown in Fig. 5a. After 3,000 cycles, there is no significant change in HER performance except for a slight potential shift. Kibsgaard reported that the slight potential shift caused by not the decline of electrocatalytic activity but rigorous H_2_ bubble formation in structure of electrodes, which ultimately results in fewer active sites for HER^19^. In contrast, unsupported MoS_x_ showed a considerable decrease in current density from 14.5 to 5.6 mA/cm^2^ after 1,000 cycles. It is believed that the excellent durability of MoS_x_/r-GO originated from the functional coupling of oxygen bridges between MoS_x_ and r-GO, leading to thermodynamic stability of the MoS_x_ particles. The XPS analysis was conducted to investigate the structural changes before and after the durability test. The atomic ratio of S to Mo after the durability test was converted from 2.6 to 2.0 based on the XPS spectrum as shown in Figure S6. Further, the BE of the deconvoluted S 2p peaks was also shifted to lower positions, as in MoS_2_. Previous studies reported that MoS_3_ is electrochemically reduced to MoS_2_ as the active species for HER^31^. However, covalent S-O and Mo-O bonds are retained after 1,000 cycles, indicating that MoS_x_ particles could be anchored on the r-GO. Therefore, we believe that the oxygen bridges might improve the stability of HER compared to MoS_3_ on multi walled carbon nanotubes with no functional coupling between the MoS_3_ and the support (88% after 500 cycles vs initial activity)^32^.

The functional coupling between MoS_x_ and r-GO was also significantly effective in preventing oxidation from affecting catalytic stability. The electrochemical oxidation test was conducted in 0.5 M H_2_SO_4_ electrolyte at positive potential. Unsupported MoS_3_ initially shows two dominant oxidation peaks at approximately 0.50 and 0.95 V as depicted in Fig. 5b. Thermodynamically unstable sulfur atoms located at edge sites are oxidized first at 0.5 V, and the rest of sulfur atoms in the basal plane are then oxidized later at nearly 0.95 V^33^. However, the oxidation potential of 0.5 MoS_x_/r-GO is positively shifted to 0.65 V (black arrow in Fig. 5c), indicating high oxidation resistance that is closely related to the stability. In addition, after electrochemical oxidation at 0.65 V, 0.5 MoS_x_/r-GO exhibits a negligible potential shift, whereas the current density of unsupported MoS_x_ decreases significantly as shown in Fig. 5d. Therefore, the novel functional coupling of oxygen could induce anchoring and oxidation-resistance effects through the strong interaction between MoS_x_ and r-GO, leading to the realization of Mo sulfide based catalysts with tremendous activity and durability.

## Discussion

In summary, we synthesized MoS_x_ anchored r-GO composite catalysts by a simple one-pot solution process at room temperature. MoS_x_ particles were covalently bonded to r-GO through oxygen functional groups, and GO was simultaneously reduced to conductive r-GO. The oxygen atoms bridged between MoS_x_ and GO play substantial roles in the fine dispersion of MoS_x_ particles, control of planar MoS_x_ growth, and increase of exposed active sulfur sites, leading to highly efficient and stable electrocatalysts for hydrogen evolution. Therefore, biaxially grown MoS_x_ anchored with r-GO could act as promising nonprecious electrocatalysts for the future hydrogen-based energy world.

## Methods

### The preparation of GO

The GO was prepared via a modified Hummers method as described in a previous report^16^. First, graphite was dispersed in sulfuric acid (133 mg/ml) by sonication and stirring. Then, KMnO_4_ was slowly added to suspension at low temperature, which was kept at 45 °C for 6 h. Then, 100 mL of distilled water and 20 mL of H_2_O_2_ were added to remove any residual oxidizing agent. The brownish mixture was washed by centrifugation. The resulting gel-like GO was freeze-dried at −45 °C for 24 h and used for the preparation of the MoS_x_/r-GO composite materials.

### The preparation of MoS_x_/r-GO composite materials

First, GO was dispersed in deionized water at a concentration of 3 mg/ml with a brief bath-sonication. Then, a specific amount of ammonium thiomolybdate (0.1, 0.3, 0.5, or 0.7 g) as a MoS_x_ precursor was separately added in 100 ml of GO dispersion with constant stirring at room temperature. Hydrochloric acid (5 ml) was slowly added to the homogeneous mixture. After gas evolution was completed, the product was centrifuged at 7000 rpm for 10 min, followed by washed using ethanol and water to remove acidic residues. Finally, the resulting gel-like MoS_x_/r-GO was freeze-dried at −45 °C for 24 h and used as the hydrogen evolution catalyst.

### Sample characterization

The crystal structure was investigated using XRD equipment (Smartlab 3, Rigaku) with a scan rate of 2 degree/min from 5 to 70 degrees. The morphologies of the prepared materials were analyzed using atomic force microscopy (AFM, Veeco, Digital Instruments Nanoscope IIIA). A sample for AFM measurement was prepared by spin-coating the catalyst dispersed in DMF at a concentration of ~1 mg/mL onto a Si wafer. The surface morphology and atomic contents of Mo, S, and C in the catalysts were analyzed using a field emission transmission electron microscope (FETEM, JEOL, JEM-2200FS) and X-ray photoelectron spectroscopy (XPS, Thermo Fisher, Multilab 2000), respectively. The TEM specimens were prepared by mixing the products in ethanol using an ultrasonic bath for 5 min, and then a drop of the suspension was placed on a copper grid. The XPS data were recorded using Al Kα radiation (hν = 1000 eV). The electrical conductivity was investigated using a four-point probe instrument (FPP-RS8, Dasol Eng.) and the film thickness of each catalyst was analyzed using a surface profiler (Alphastep IQ, KLA Tencor).

### Electrochemical analysis

First, 15 mg of each MoS_x_/r-GO composite powder was dispersed in a mixture of 1000 μl of DMF and 100 μL of Nafion with a brief sonication. Then, 8 μL of the prepared sample was deposited on glassy carbon electrode stand tried at at 50 °C. Linear sweep voltammetry using a potentiostat with a scan rate of 5 mVs^−1^ was conducted in 0.5 M H_2_SO_4_ electrolyte using an Ag/AgCl electrode as the reference electrode and a platinum wire as the counter electrode.

### Calculation of electrochemical active sites and TOF

The oxidation peak at lower potential indicated the oxidation potential of edge area of MoS_x_ to MoO_2_ as shown in Fig. 5c. Thus, total current of edge oxidation peak was used to calculate the electrochemical active sites. The following equations were used to calculate the active sites and TOF.


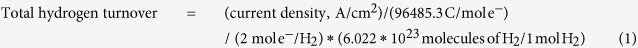



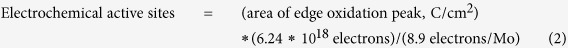



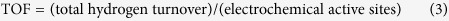


we assumed that the average number of electrons for each Mo oxidation is approximately 8.9 electrons^32^.

## Additional Information

**How to cite this article**: Lee, C.-H. *et al*. Bi-axial grown amorphous MoS_x_ bridged with oxygen on r-GO as a superior stable and efficient nonprecious catalyst for hydrogen evolution. *Sci. Rep.* **7**, 41190; doi: 10.1038/srep41190 (2017).

**Publisher's note:** Springer Nature remains neutral with regard to jurisdictional claims in published maps and institutional affiliations.

## Supplementary Material

Supporting Information
