# Supplementary material for: Bi-axial grown amorphous MoSx bridged with oxygen on r-GO as a superior stable and efficient nonprecious catalyst for hydrogen evolution
Source: Sci Rep. 2017 Jan 20;7:41190. doi: 10.1038/srep41190 (PMC5247739; doi:10.1038/srep41190)
Supplement: Supporting Information [file srep41190-s1.doc]

***Supplementary Information***

***Bi-axial grown amorphous MoS_x_ bridged with oxygen on r-GO as a superior stable and efficient nonprecious catalyst for hydrogen evolution***

Cheol-Ho Lee^a,b,†^, Jin-Mun Yun^c,†^, Sungho Lee^a^, Seong Mu Jo^a^, KwangSup Eom^d^, Doh C. Lee^b,*^, Han-Ik Joh^a^,[[1]](#footnote-2), and Thomas F. Fuller^d^

*^a^ Carbon Convergence Materials Research Center, Institute of Advanced Composite Materials, Korea Institute of Science and Technology (KIST), chudong-ro 92, Bongdong-eup, Wanju, Jeollabukdo 55324, Republic of Korea.*

*^b^ Department of Chemical and Biomolecular Engineering (BK21+ Program), KAIST Institute for the Nanocentury, Korea Advanced Institute of Science and Technology (KAIST), Daejeon 34141, Republic of Korea.*

*^c^ Radiation Research Division for Industry and Environment, Korea Atomic Energy Research Institute (KAERI), Geumgu-gil 29, Jeongeup-si, Jeollabuk-do 56212, Republic of Korea.*

*^d^ School of Chemical & Biomolecular Engineering, Georgia Institute of Technology, Atlanta, Georgia 30332, USA.*

**
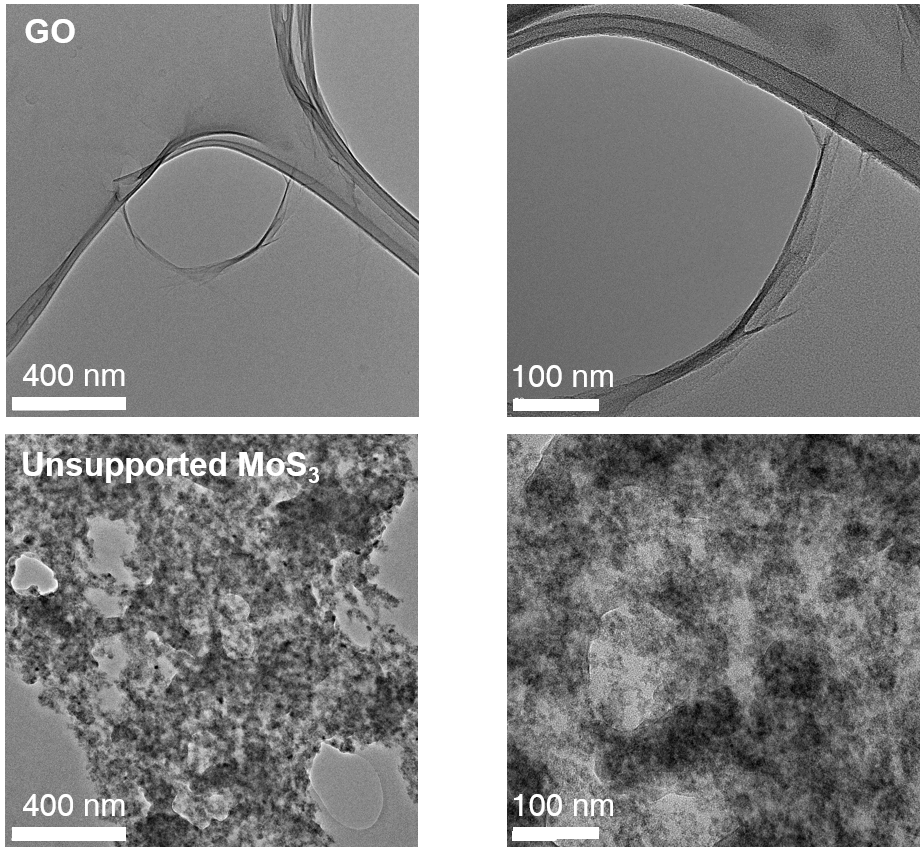
**

**Figure S1. TEM images of GO (top) and unsupported MoS_3_ (bottom).**

**
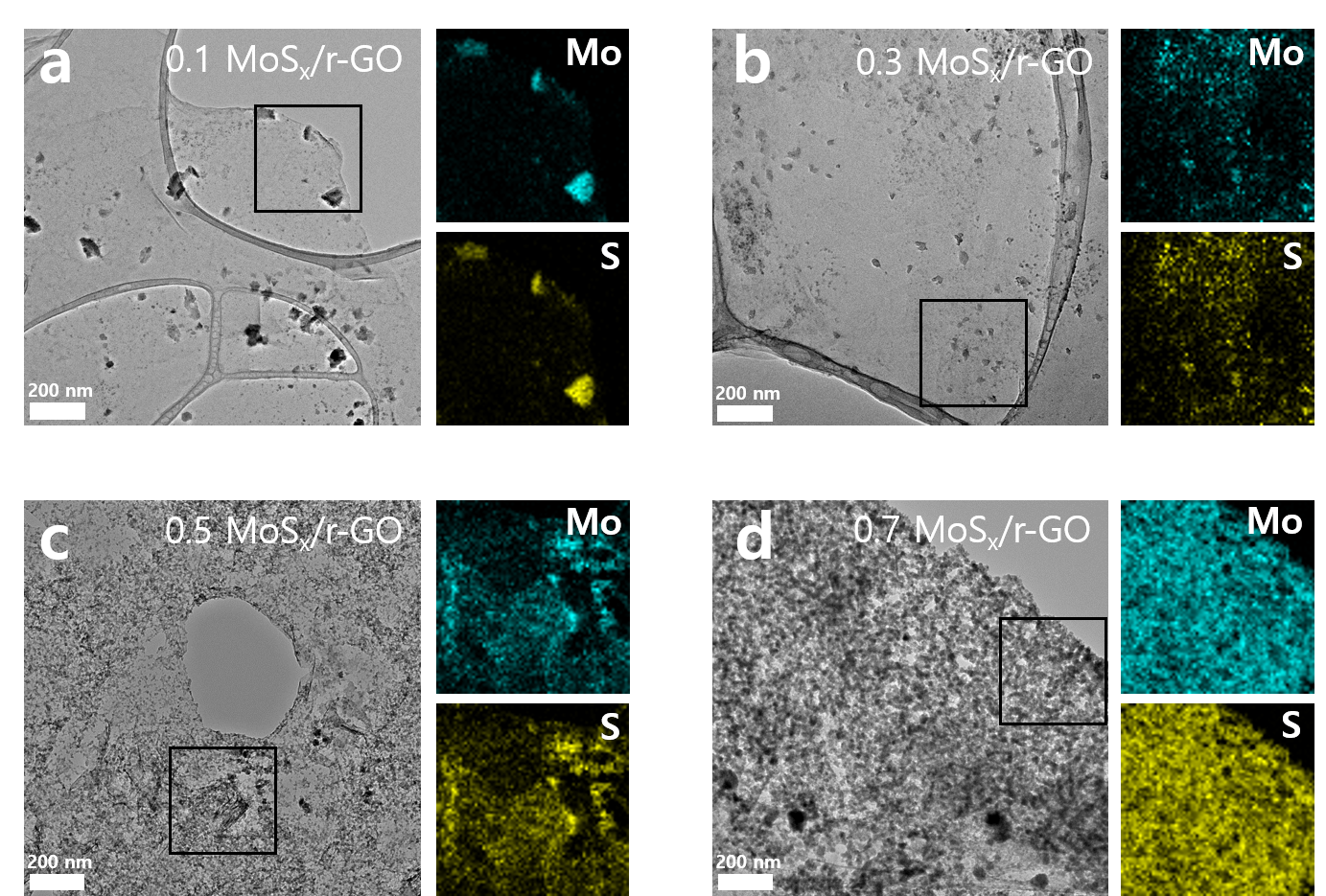
**

**Figure S2. TEM images and EDS mapping analysis of MoS_x_/r-GO composites. Hollow and black-lined box in TEM images indicate area of EDS measurement.**

**
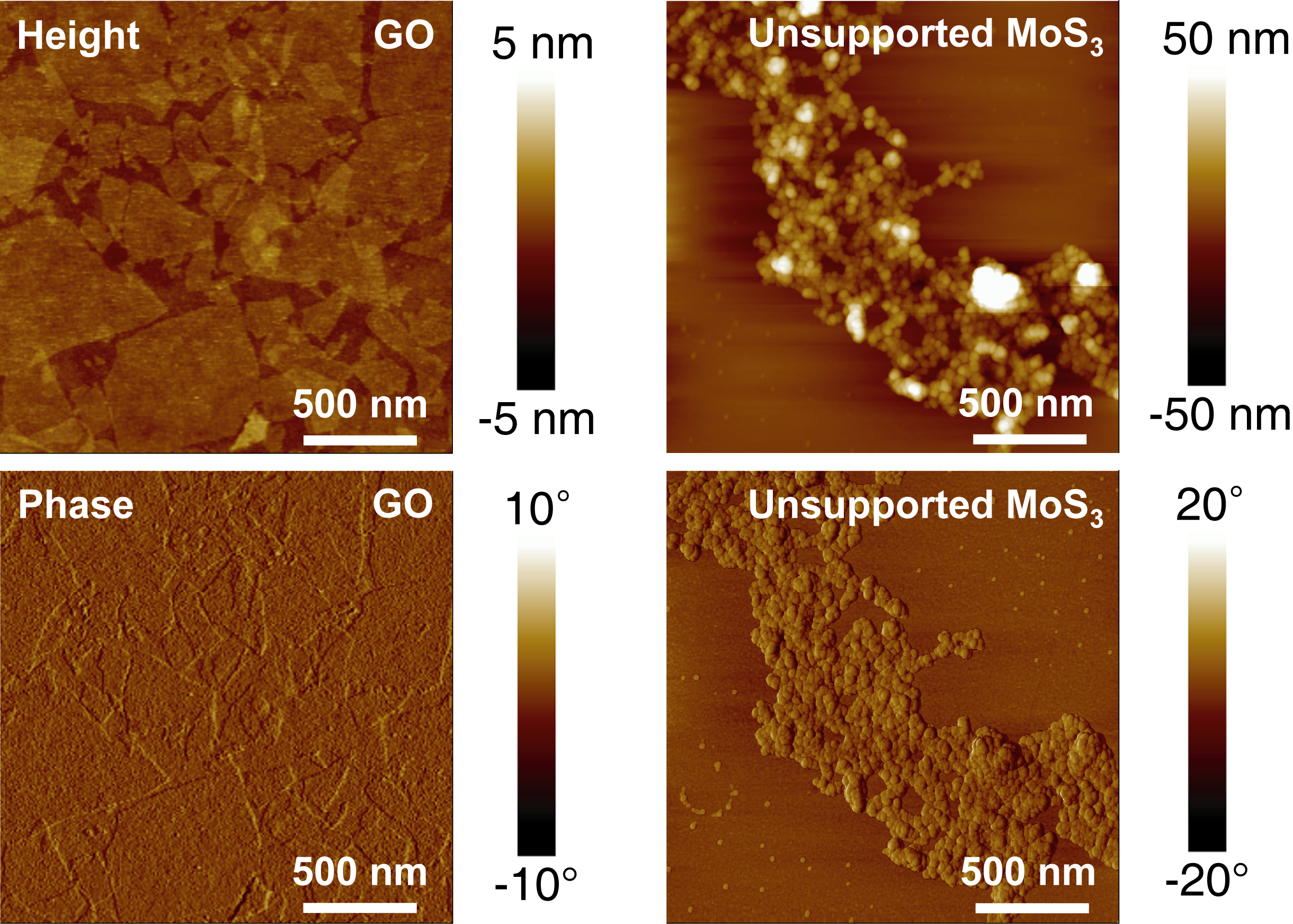
**

**Figure S3. AFM height and phase images of GO and unsupported MoS_3_.**

**
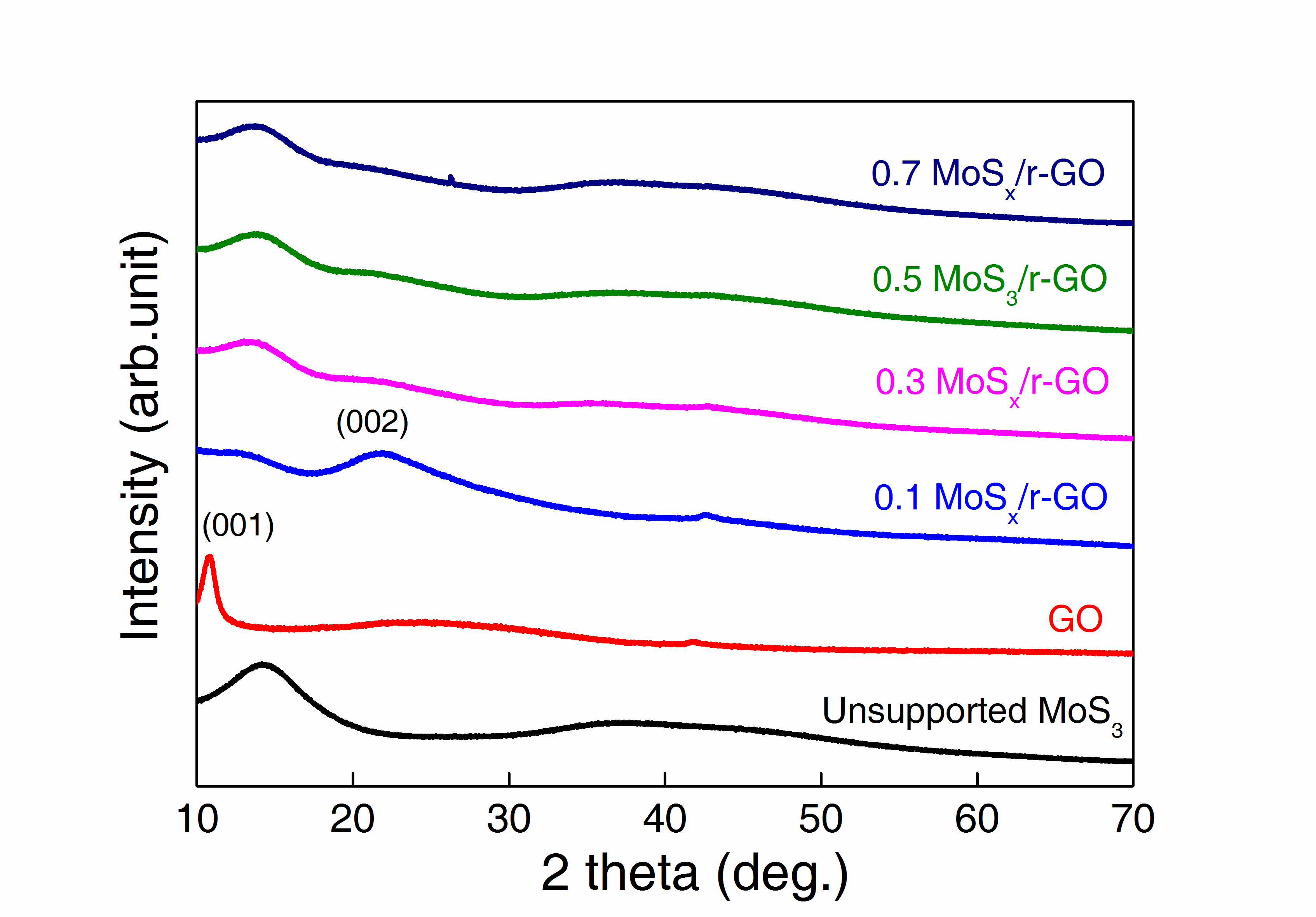
**

**Figure S4. XRD patterns of GO, unsupported MoS_3_, and MoS_x_/r-GO composites.**

**
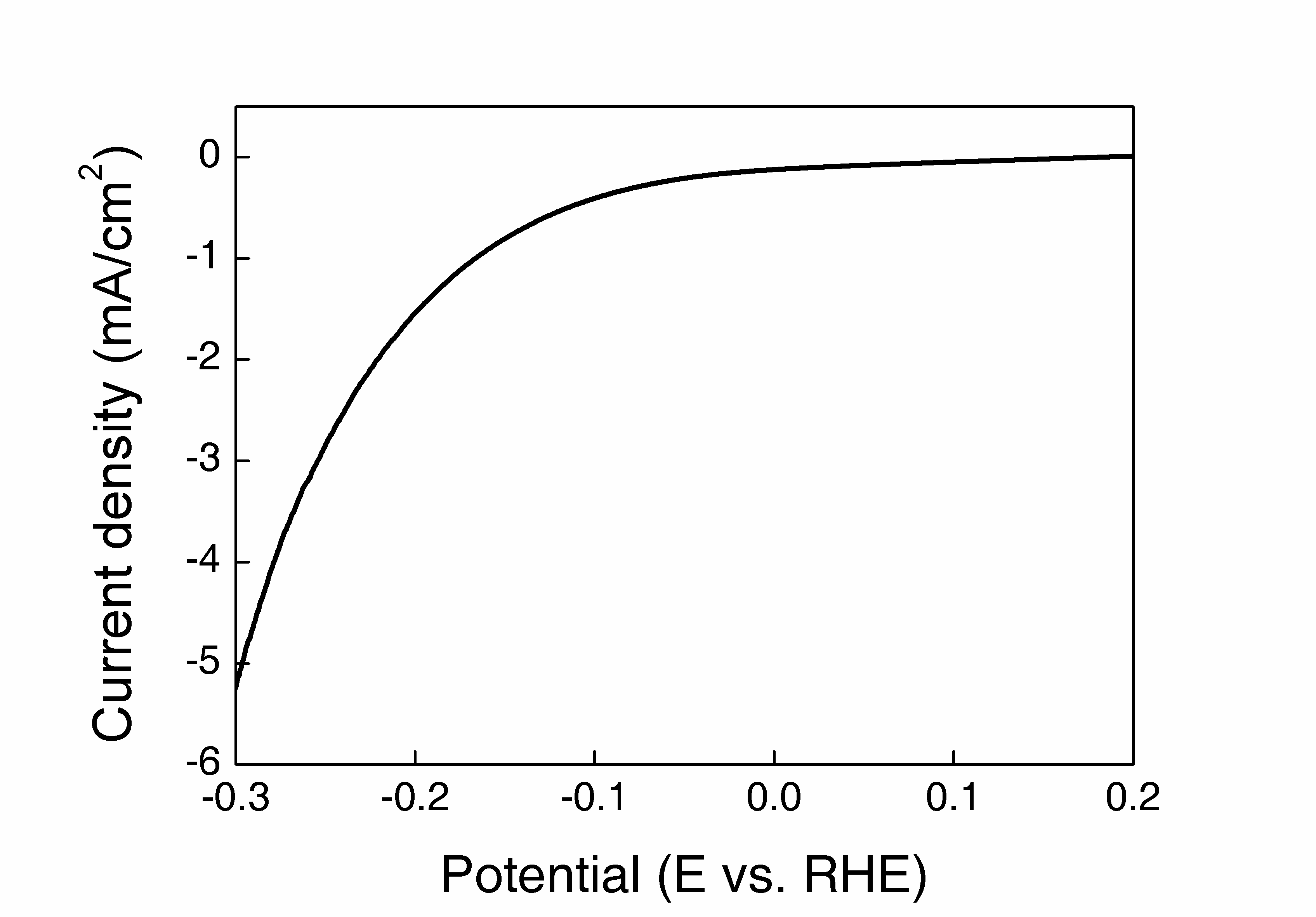
**

**Figure S5. Electrocatalytic HER activity of pristine r-GO reduced using phenylhydrazine.**

**
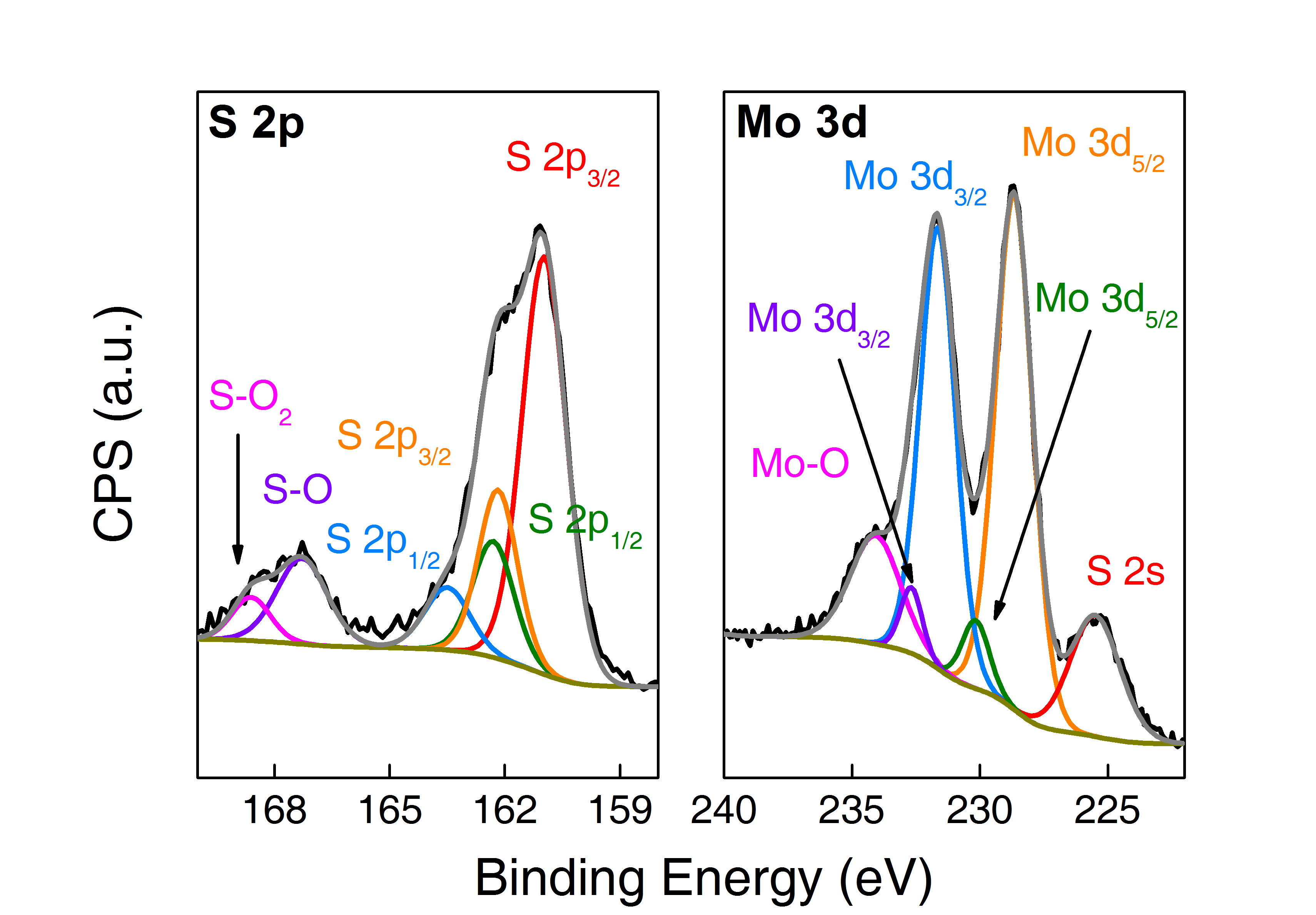
**

**Figure S6. High-resolution XPS analysis of 0.5 MoS_x_/r-GO after 1,000 cycles with deconvoluted S 2p and Mo 3d peaks.**

1. ^†^ C. -H. Lee and J. –M. Yun contributed equally to this work.

   ? Corresponding Author: Tel/Fax: +82 63 219 8137/+82 63 219 8139 (H. I. Joh) and +82 42 350 3937 (D. Lee), E-mail address: [hijoh@kist.re.kr](mailto:hijoh@kist.re.kr) (H. I. Joh) and [dclee@kaist.edu](mailto:dclee@kaist.edu) (D. Lee) [↑](#footnote-ref-2)
